# A Toothache That Was a Stroke: Acute Right Parietal Infarction Presenting As Isolated Left-Sided Odontogenic-Like Pain

**DOI:** 10.7759/cureus.114141

**Published:** 2026-08-07

**Authors:** Ereida Rraklli, Arlinda Abazaj, Kjanda Elpenoria, Almira Isufi

**Affiliations:** 1 Neurology, Berat Regional Hospital, Berat, ALB; 2 Neurology, Fier Memorial Regional Hospital, Fier, ALB; 3 Dentistry, University of Medicine, Tirana, Tirana, ALB; 4 Endodontics, Boston University Henry M. Goldman School of Dental Medicine, Boston, USA; 5 Health Sciences, UniCamillus - Saint Camillus International University of Health and Medical Sciences, Rome, ITA

**Keywords:** acute ischemic stroke, central neuropathic pain, diffusion-weighted imaging, odontogenic-like pain, orofacial pain, parietal infarction, toothache, trigeminal pain pathways

## Abstract

Pain as a presenting symptom of acute ischemic stroke is uncommon and may delay diagnosis when typical focal neurological deficits are absent. A 52-year-old man with arterial hypertension and a history of tobacco and alcohol use presented to the ED with sudden, severe left-sided toothache-like pain perceived as arising from the teeth. He had no recent dental procedure, trauma, fever, or systemic infectious symptoms. An oral and dental examination revealed no gingival swelling, facial cellulitis, dental abscess, percussion tenderness, or other convincing odontogenic cause. Neurological examination revealed no facial weakness, dysarthria, aphasia, hemiparesis, limb ataxia, visual field deficit, cranial nerve abnormality, or definite objective sensory loss. Non-contrast CT of the brain excluded intracranial hemorrhage. Brain MRI with diffusion-weighted imaging demonstrated an acute right parietal cortico-subcortical ischemic lesion. The patient was treated with aspirin for secondary stroke prevention, and pregabalin was prescribed for the symptomatic management of presumed central neuropathic pain. The odontogenic-like pain improved substantially during follow-up. This case demonstrates that acute parietal ischemic stroke may present as isolated contralateral toothache-like pain. Acute, severe dental or orofacial pain without a clear odontogenic explanation should prompt consideration of a neurological cause, particularly in patients with vascular risk factors. Diffusion-weighted MRI may be necessary when clinical suspicion of acute ischemic stroke persists despite an otherwise unremarkable neurological examination.

## Introduction

Acute ischemic stroke usually presents with sudden focal neurological deficits, including hemiparesis, aphasia, dysarthria, visual field loss, sensory impairment, or ataxia. Pain as the first or only manifestation of stroke is uncommon and may lead to diagnostic delay, particularly when the pain resembles a common non-neurological disorder.

Orofacial and dental-like pain caused by stroke has been described mainly in association with brainstem lesions, which involve the trigeminal nuclei, trigeminal root entry zones, and ascending sensory pathways [1-3]. Pontine and lateral medullary infarctions may produce facial pain or pain mimicking trigeminal neuralgia through the involvement of trigeminal sensory pathways [2,3]. By contrast, cortical ischemic stroke presenting as isolated odontogenic-like pain is much less frequently reported.

We describe a 52-year-old man with an acute right parietal cortico-subcortical infarction whose only presenting symptom was sudden, severe left-sided toothache-like pain. We also review the relevant literature on painful stroke presentations, stroke-related dental or facial pain, cortical pain mechanisms, and the diagnostic implications of this unusual presentation.

## Case presentation

A 52-year-old man presented to the ED because of the abrupt onset of severe left-sided dental pain. The pain was intense and continuous and was perceived by the patient as originating from the teeth. His medical history was notable for arterial hypertension and a previous ischemic stroke in 2018, from which he had made a complete neurological recovery without residual deficits. He also reported moderate tobacco and alcohol use. He had no known history of diabetes mellitus or transient ischemic attack. There was no reported head trauma, fever, recent dental procedure, facial swelling, or systemic infectious symptoms at presentation (Table 1).

**Table 1:** Hematologic and biochemical laboratory findings. ALT: Alanine aminotransferase; AST: Aspartate aminotransferase; F: Female; HDL: High-density lipoprotein; LDL: Low-density lipoprotein; M: Male; MCH: Mean corpuscular hemoglobin; MCHC: Mean corpuscular hemoglobin concentration; MCV: Mean corpuscular volume; MID: Mixed-cell count; MPV: Mean platelet volume; PCT: Plateletcrit; RDW-CV: Red cell distribution width–coefficient of variation.

Test	Result	Unit	Reference Range
WBC	4.9	× 10³/µL	4.00-11.00
Lymphocytes	1	× 10³/µL	1.1-3.6
MID	0.4	× 10³/µL	0.2-1.2
Granulocytes	3.5	× 10³/µL	1.9-7.9
Lymphocytes (%)	21.3	%	14.1-52.8
MID (%)	7.7	%	3.2-17.7
Granulocytes (%)	71	%	39.6-78.4
RBC	4.65	× 10⁶/µL	3.85-5.78
Hemoglobin	12.8	g/dL	12.0-17.2
MCV	78.9	fL	78.0-96.0
Hematocrit	35.4	%	34.8-50.9
MCH	27.2	pg	26.4-33.2
MCHC	32.2	g/dL	31.8-36.7
RDW-CV	16.8	%	13.1-24.6
Platelets	173	× 10³/µL	150-400
MPV	7.8	fL	7.0-11.5
PCT	0.13	%	0.07-0.49
ESR	21	mm/h	0-20
Glucose	99.2	mg/dL	70-110
Urea	34.3	mg/dL	10-50
Creatinine	0.91	mg/dL	0.6-1.3
ALT	8.2	U/L	<45 (M), <34 (F)
AST	19.2	U/L	<35 (M), <31 (F)
Total bilirubin	0.93	mg/dL	0.1-1.2
Total cholesterol	195	mg/dL	<200
Triglycerides	119	mg/dL	<150
HDL cholesterol	53.91	mg/dL	35-80
LDL cholesterol	117.3	mg/dL	0-150

A focused oral and dental examination did not reveal gingival swelling, facial cellulitis, dental abscess, visible oral infection, or percussion tenderness. There were no clinical findings strongly supporting acute pulpitis, a dental abscess, or another local odontogenic cause.

On neurological examination, the patient was alert and oriented. There was no facial weakness, dysarthria, aphasia, hemiparesis, limb ataxia, visual field deficit, or clinically evident cranial nerve abnormality. Sensory examination did not demonstrate a clear objective hemisensory deficit. The pain was left-sided and was not associated with typical triggers suggestive of classical trigeminal neuralgia.

**Figure 1:**
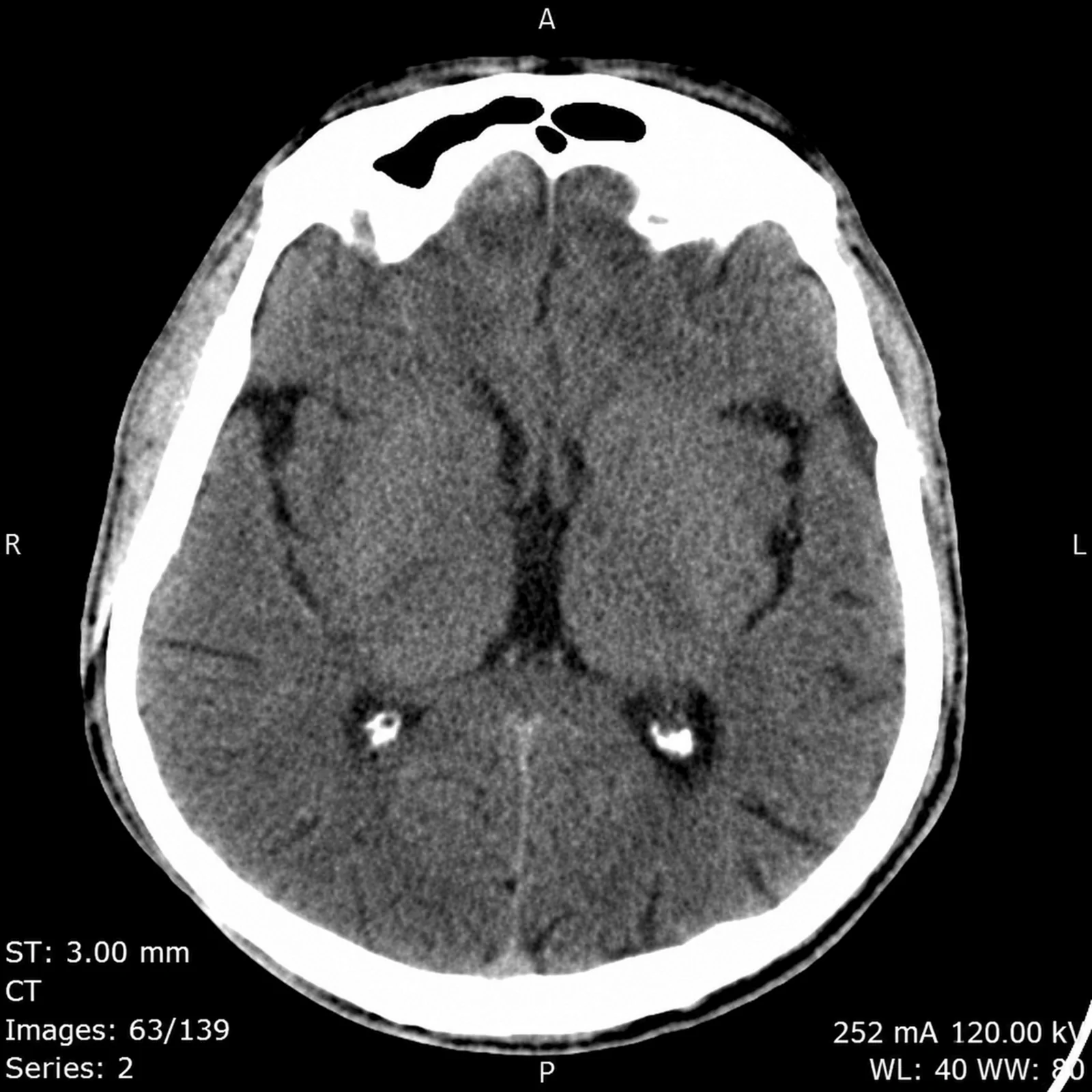
Axial non-contrast computed tomography image of the head. Representative axial non-contrast computed tomography image showing no acute intracranial hemorrhage or significant mass effect.

Because of the abrupt onset and severity of the pain, the absence of a convincing dental explanation, and the patient’s vascular risk profile, urgent brain imaging was performed. Non-contrast CT of the head showed no intracranial hemorrhage (Figure 1).

**Figure 2:**
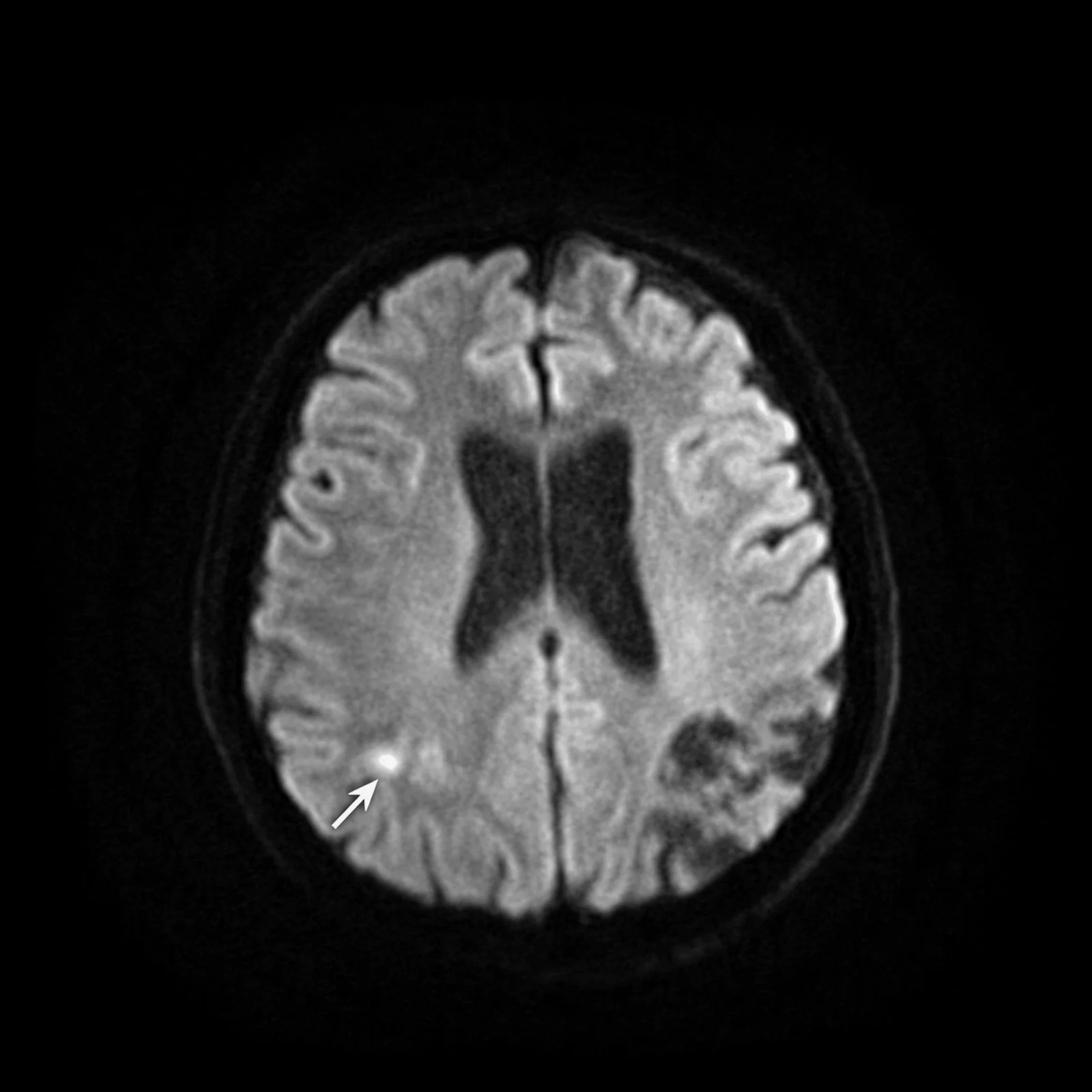
Axial diffusion-weighted MRI. Axial diffusion-weighted MRI showing a small focal hyperintense lesion in the right parietal cortico-subcortical region (arrow), consistent with an acute ischemic infarct.

Brain MRI with diffusion-weighted imaging demonstrated a focal acute ischemic lesion in the right parietal cortico-subcortical region, compatible with an acute ischemic stroke (Figure 2).

The patient was treated with aspirin for secondary stroke prevention. Pregabalin was added for the symptomatic management of presumed central neuropathic pain. During follow-up, the left-sided odontogenic-like pain improved significantly without dental intervention. Electroneuromyography was not performed because there were no clinical signs suggesting peripheral trigeminal neuropathy, facial nerve involvement, or neuromuscular disease. The abrupt onset and diffusion-weighted MRI findings supported a central ischemic mechanism.

## Discussion

This case is clinically important because the presenting complaint closely mimicked an odontogenic disorder, yet neuroimaging confirmed an acute ischemic stroke. The absence of classical focal neurological deficits could have led to the attribution of the pain to a dental or maxillofacial cause. However, the sudden onset and severity of the pain, the patient’s vascular risk profile, and the absence of local dental pathology supported urgent neurological evaluation.

The second important feature is the lesion location. Most previously reported cases of stroke-associated facial or dental-like pain involve the brainstem, especially the pons or medulla, where important trigeminal sensory structures are located [2,3]. In our patient, the infarct was supratentorial and localized to the right parietal cortico-subcortical region. The pain was contralateral to the lesion and affected the left dental/orofacial region.

Literature review

Painful stroke presentations are uncommon but well recognized. Bayat M et al. reviewed atypical painful stroke presentations and emphasized that pain may occasionally be the initial manifestation of ischemic stroke, contributing to misdiagnosis or delayed recognition. Central post-stroke pain is more often described as a delayed complication, but acute or early pain presentations have been reported [1].

Dental-like pain caused by stroke has been most clearly described in brainstem infarction. Goel R et al. reported a patient with acute dental pain and a normal dental examination who was later found to have a pontine infarct near the trigeminal root entry zone [2]. Shi Y et al. described cases of acute ischemic brainstem stroke presenting with pain mimicking trigeminal neuralgia [3]. These cases support the concept that ischemic injury affecting the trigeminal nuclei, root entry zones, or ascending trigeminal pathways can produce pain perceived in the dental or facial territory.

Parietal stroke presenting primarily with pain is rarer. Saucedo MA et al. reported pain as the first manifestation of an acute ischemic parietal stroke, but the pain involved the contralateral limb rather than an isolated dental distribution [4]. Our case differs because the symptom was isolated left-sided odontogenic-like pain, while the infarct was located in the right parietal cortico-subcortical region.

Experimental and functional neuroimaging studies provide a plausible mechanism. Tooth pain activates cortical pain networks, including the primary and secondary somatosensory cortices, the insula, and the cingulate cortex [5]. Functional MRI studies have demonstrated the representation of oral structures within the human somatosensory cortex [6]. Therefore, a parietal infarct may disrupt cortical sensory integration and generate pain misperceived as arising from the teeth.

Pathophysiological considerations

The patient’s left-sided toothache-like pain may be explained by disruption of central sensory processing within the right parietal region. Trigeminal nociceptive input from the teeth and orofacial structures ascends through brainstem trigeminal pathways and thalamocortical projections to cortical regions involved in sensory-discriminative and affective pain processing. These regions include the primary and secondary somatosensory cortices, the insula, and the anterior cingulate cortex [5-7].

The parietal lobe, particularly the postcentral gyrus and adjacent somatosensory association areas, plays a key role in the localization, interpretation, and integration of sensory input. Ischemia in this region may disturb the normal processing of trigeminal nociceptive information. As a result, abnormal cortical activity may be perceived as pain arising from a peripheral structure, such as the teeth, despite the absence of dental pathology.

This mechanism differs from peripheral trigeminal neuropathy. In peripheral trigeminal neuropathy, pain is usually related to injury, compression, inflammation, or dysfunction of the trigeminal nerve or its branches. In this case, there were no clinical signs of peripheral trigeminal involvement, and the temporal relationship between the abrupt onset of pain and diffusion-weighted MRI evidence of a right parietal infarction favored a central ischemic mechanism.

Differential diagnosis

The main diagnostic challenge was distinguishing a common odontogenic disorder from a rare central neurological cause. The differential diagnosis included acute pulpitis, dental abscess, periodontal disease, cracked tooth syndrome, trigeminal neuralgia, trigeminal neuropathy, temporomandibular disorder, sinus disease, herpes zoster, migraine-related facial pain, and central neuropathic pain due to acute ischemic stroke.

Diagnostic and clinical implications

This case emphasizes several practical points. First, dental pain without a convincing dental explanation should not automatically be treated as a primary odontogenic disorder, particularly when the onset is abrupt and the patient has vascular risk factors. Second, a normal non-contrast CT scan does not exclude early ischemic stroke. Non-contrast CT is commonly used to exclude intracranial hemorrhage, whereas diffusion-weighted MRI is more sensitive for detecting acute ischemia [8]. Third, collaboration among dental, maxillofacial, emergency medicine, and neurology teams may prevent unnecessary dental procedures and delayed stroke diagnosis.

## Conclusions

An acute right parietal ischemic stroke may rarely present as isolated contralateral odontogenic-like pain. In this patient, sudden, severe left-sided toothache-like pain occurred without dental pathology or objective focal neurological deficits, but diffusion-weighted MRI confirmed an acute right parietal cortico-subcortical infarction. This case expands the clinical spectrum of painful stroke presentations and highlights the need to consider stroke in patients with abrupt, unexplained dental or orofacial pain, especially when vascular risk factors are present.
